# Thymosin *β*4 Improves Differentiation and Vascularization of EHTs

**DOI:** 10.1155/2017/6848271

**Published:** 2017-01-16

**Authors:** Tilman Ziegler, Rabea Hinkel, Andrea Stöhr, Thomas Eschenhagen, Karl-Ludwig Laugwitz, Ferdinand le Noble, Robert David, Arne Hansen, Christian Kupatt

**Affiliations:** ^1^I. Medizinische Klinik und Poliklinik, Klinikum rechts der Isar, Munich, Germany; ^2^German Center for Cardiovascular Research (DZHK), Partner Site Munich Heart Alliance, Munich, Germany; ^3^Institute for Cardiovascular Prevention, Ludwig Maximilians University, Munich, Germany; ^4^Institut für Experimentelle Pharmakologie und Toxikologie, Universitätsklinikum Hamburg-Eppendorf, Hamburg, Germany; ^5^Karlsruher Institut für Technologie, Karlsruhe, Germany; ^6^Department of Cardiac Surgery, Reference and Translation Center for Cardiac Stem Cell Therapy (RTC), University of Rostock, Rostock, Germany; ^7^German Center for Cardiovascular Research (DZHK), Partner Site Hamburg/Kiel/Lübeck, Hamburg, Germany

## Abstract

Induced pluripotent stem cells (iPSC) constitute a powerful tool to study cardiac physiology and represents a promising treatment strategy to tackle cardiac disease. However, iPSCs remain relatively immature after differentiation. Additionally, engineered heart tissue (EHT) has been investigated as a therapy option in preclinical disease models with promising results, although their vascularization and functionality leave room for improvement. Thymosin *β*4 (T*β*4) has been shown to promote the differentiation of progenitor cell lines to cardiomyocytes while it also induces angiogenic sprouting and vascular maturation. We examined the potential impact of T*β*4 to enhance maturation of cardiomyocytes from iPSCs. Assessing the expression of transcription factors associated with cardiac differentiation, we were able to demonstrate the increased generation of cells displaying cardiomyocyte characteristics in vitro. Furthermore, we demonstrated, in a zebrafish model of embryonic vascular development, that T*β*4 is crucial for the proper execution of lymphatic and angiogenic vessel sprouting. Finally, utilizing T*β*4-transduced EHTs generated from mice genetically engineered to label endothelial cells in vitro, we show that treatment with T*β*4 promotes vascularization and contractility in EHTs, highlighting T*β*4 as a growth factor improving the formation of cardiomyocytes from iPSC and enhancing the performance of EHTs generated from neonatal cardiomyocytes.

## 1. Introduction

Engineered heart tissue generated from neonatal cardiomyocytes has been a useful tool to study cardiac physiology and presents a promising treatment option for patients with heart failure or excessive scar tissue formation after myocardial infarction, which has been demonstrated in preclinical animal models of myocardial replacement therapy [1, 2]. However, major obstacles in generating functioning EHT-grafts of sufficient size still remain. For instance, while grafts can generate primitive vascular networks [3], those vessels typically are unable to support EHTs of increasing thickness [4]. Additionally, myocardial-grafts derived from neonatal cardiomyocytes lack the level of maturity seen in adult myocardium. On the one hand, those EHTs do display features of fully matured, adult cardiomyocytes, such as the expression of contractile proteins, their organization into sarcomeric structures, and the capability to mimic basic cardiomyocyte physiology [5–7]. However, thusly generated engineered heart tissue grafts still have a higher ratio of fetal myosin heavy chain and are unable to generate comparable force [8], which reflects their relative immaturity.

Thymosin *β*4 (T*β*4), although first described as a G-actin sequestering peptide regulating cytoskeletal rearrangement and cellular motility [9], has recently been shown to possess additional functions during cell fate determination and angiogenesis. Thus, it was demonstrated that T*β*4 in mouse models of myocardial ischemia promotes the proliferation of mesenchymal stem cells, improving cardiac function [9], and advances the transdifferentiation of epicardial progenitors into integrated cardiomyocytes [10]. Furthermore, T*β*4 was shown to facilitate angiogenic sprouting, by activating SRF target genes via the nuclear translocation of MRTF-A [11], and to foster the attachment of mural cells to endothelial cell tubes, improving the functionality of vessels [12, 13].

On account of Thymosin *β*4 promoting cardiomyocyte differentiation and improving tissue supply with functioning vasculature, we investigated the effect of T*β*4 on the differentiation of iPSCs to myocardial cells and its impact on the quality of engineered heart tissues.

## 2. Materials and Methods

### 2.1. Generation of Induced Pluripotent Stem Cells

Induced pluripotent stem cells derived from murine embryonic fibroblasts were kindly provided by Ulrich Martin [14]. Cells were cultured on mitotically inactivated murine embryonic fibroblasts (MEFs; 50 000 cells/cm^2^) in 6-well plates. The culture medium was composed of Dulbecco's modified Eagle's medium (Invitrogen, Carlsbad, Calif) with 15% fetal calf serum (Thermo Fisher Scientific Germany), 0.2 mmol/L l-glutamine (Invitrogen), 0.1 mmol/L *β*-mercaptoethanol (Invitrogen), 0.1 mmol/L nonessential amino acid stock (Invitrogen), and 0.1% human leukemia inhibitory factor-conditioned medium, which had been produced by transient transfection of a human leukemia inhibitory factor expression plasmid into human embryonic kidney 293 cells. To initiate embryoid body formation, cells were cultured in the “hanging drop” technique with 600 cells/20 *μ*L of differentiation medium, consisting of Iscove's modified Dulbecco's medium with 15% FCS, 0.2 mmol/L l-glutamine, 0.1 mmol/L *β*-mercaptoethanol, and 0.1 mmol/L nonessential amino acid stock. After 3 days of differentiation EBs were transferred onto nonadherent 1% agarose-coated 96-wells, followed by a transfer of 10 EBs per well on a 0.1% gelatin-coated 6-well culture dish.

### 2.2. Vascular Patterning in Zebrafish

Embryos and adult zebrafish were raised and maintained under standard conditions. The transgenic zebrafish line Tg(fli1a:EGFP)^y1^ was used as previously described [15]. Morpholinos, used to knockdown tmsb-like expression and control morpholinos, were injected into embryos at the 1 to 2 cell stage (15 ng/embryo). The developing vasculature was examined using the Leica SP5 confocal microscope.

### 2.3. Flow Cytometry

Induced pluripotent stem cells were trypsinized, washed in cold PBS, and fixed for 20 minutes at RT in 3.7% formaldehyde. Cells were permeabilized with 0.4% Triton X-100 in PBS and subsequently incubated with antibodies as indicated for 2 hours at 37°C in 0.4% Triton X-100 in PBS. Stainings were performed with antibodies against cardiac Troponin I (BD Pharmingen, San Diego, US), *α*MHC (BD Pharmingen, San Diego, US), and *α*-actinin (Sigma Aldrich, St. Louis, US). A FITC conjugated IgG antibody served as a control. After washing, cells were analyzed on a Coulter Epics XL-MCLTM flow cytometer (Beckman Coulter, Krefeld, Germany).

### 2.4. Quantitative Real-Time PCR

RNA from iPSCs was isolated at timepoints indicated and reverse-transcribed into cDNA. qPCR was performed on a iQ-cycler (Bio-Rad, Munich, Germany) with SYBR-Green Supermix (Bio-Rad, Munich, Germany). For qPCR, the following primers were used: Brachyury (T): upper: 5′ ACCCAGCTCTAAGGAACCAC 3′; lower: 5′ ACTCCGAGGCTAGACCAGTT 3′; Eomes: upper: 5′ CTCTAGACTCCAGCGACTCC 3′; lower: 5′ GCCTTTGGAGGTGTCTTTAC 3′; Mesp1: upper: 5′ CAGTACGCAGAAACAGCATC 3′; lower: 5′ GGTTTCTAGAAGAGCCAGCA 3′; NKX-2.5: upper: 5′ CATTTACCCGGGAGCCTACG 3′; lower: 5′GCTTTCCGTCGCCGCCGTGC 3′; ANF: upper: 5′ GCAAATCCTGTGTACAGTGC 3′; lower: 5′ CAGGTGGTCTAGCAGGTTCT 3′; CX43: upper: 5′ CTGTCGGTGCTCTTCATTTTC 3′; lower: 5′ GTGGGCACAGACACGAATATG 3′.

### 2.5. Generation of EHTs

Neonatal mouse heart cells were isolated from Cdh5-CreERT2 × Rosa26-LacZ mice, expressing LacZ in endothelial cells as described previously [16]. Cells were mixed with fibrinogen (1 mg/mL, Sigma, F4753), medium (DMEM), Matrigel (100 *μ*L/mL, BD Bioscience, 356235), and thrombin (100 U/mL, Sigma, T7513) and subsequently casted into strip-format molds in agarose (dimensions 12 × 3 × 3 mm) with pairs of elastic silicone posts placed from above. After fibrinogen polymerization (2 hours, 37°C, 7% CO_2_), mold racks were transferred to a new 24-well plate and EHTs were cultured with DMEM (Biochrom F0415), supplemented with 10% horse serum (Gibco 26050), 2% chick embryo extract, 1% penicillin/streptomycin (Gibco 15140), insulin (10 *μ*g/mL, Sigma I9278), tranexamic acid (400 *μ*mol/L, Sigma 857653), and aprotinin (33 *μ*g/mL, Sigma A1153).

### 2.6. Skinfold Chamber

BALB/c nude mice were anaesthetized with an intraperitoneal injection of midazolam, medetomidine, and fentanyl and placed on a warming platform. After hair removal and fixation of the back skin via silk sutures, two openings at the base of the skinfold were prepared through which the connecting screws of the chamber were passed. The observation window was marked and one skin layer was removed. Subsequently, the previously generated EHT-grafts were sutured into the observation window and sealed with a cover glass kept in place by a snap ring. After fixing the second half of the chamber, anaesthesia was antagonized with atipamezole, flumazenil, and naloxone and mice were placed in individual cages until further use. For imaging of angiogenic sprouting derived from the EHT-grafts, whole mount X-Gal staining from Cdh5-CreERT2 × Rosa26-LacZ derived EHTs was performed and subsequently imaged via light microscopy.

### 2.7. Statistical Analysis

Data are given as mean ± SEM. Differences among several groups were tested using ANOVA and the Student Newman Keuls post hoc analysis. A *p* value of < 0.05 was considered statistically significant. All data were assessed using the SPSS software package (version 20.0; http://www.spss.com). Sample sizes are provided in the figure legends.

## 3. Results and Discussion

### 3.1. Thymosin *β*4 Drives Cardiac Differentiation in Induced Pluripotent Stem Cells

**Figure 1:**
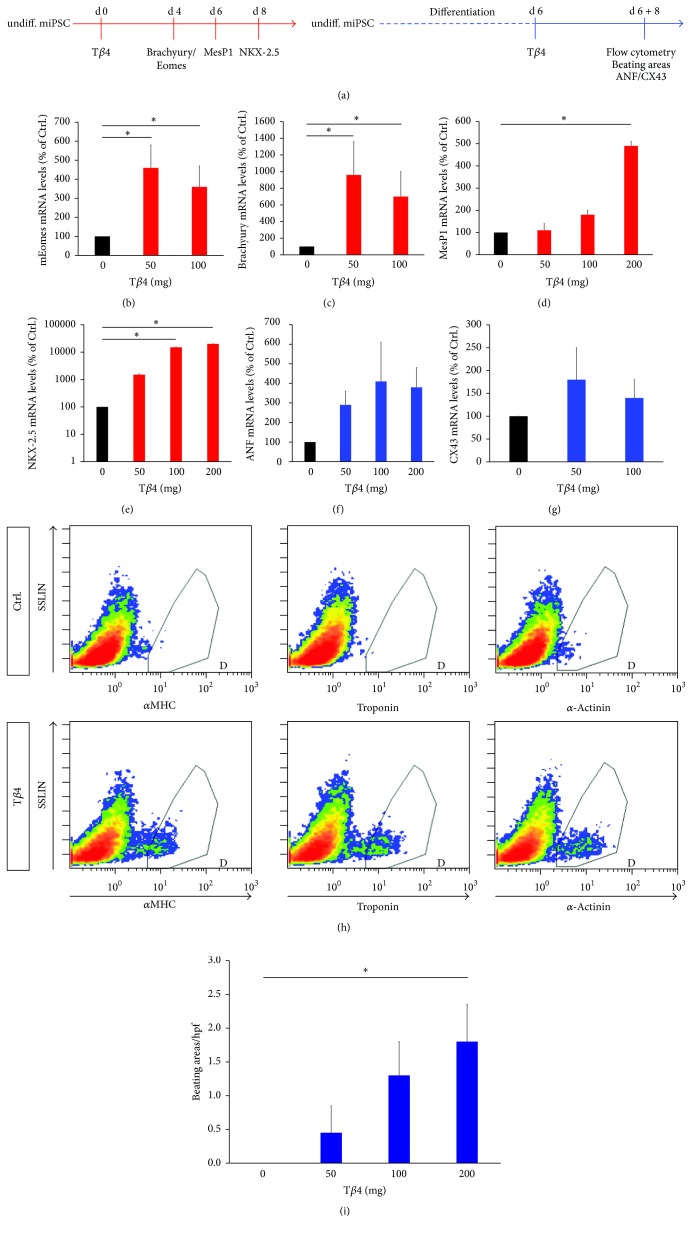
Thymosin *β*4 promotes the generation of cardiomyocytes from murine induced pluripotent stem cells. (a) Undifferentiated iPSCs were treated with T*β*4-peptide and expression of transcription factor encoding mRNAs was measured 4 (Eomes, Brachyury (T)), 6 (Mesp1), or 8 (NKX-2.5) days after T*β*4 treatment (red). In another approach, murine iPSCs were differentiated for 6 days and treated with T*β*4-peptide. 8 days after T*β*4 treatment, expression levels of cardiomyocyte markers (ANF, CX43) were measured and flow cytometric assessment of cardiac specific proteins (*α*MHC, Troponin, and *α*-actinin) was performed. Furthermore, the amount of beating areas was measured (blue). (b–g) Relative expression levels of transcripts as indicated ((b–e) without prior differentiation of iPSCs, (f and g) with 6 days of prior differentiation), measured via quantitative real-time PCR, reveal that T*β*4 induces the expression of transcription factors regulating the differentiation of stem cells to cardiomyocytes via mesenchymal and cardiac progenitor stages and the expression of cardiac specific transcripts. (h) Flow cytometry after intracellular staining for *α*MHC, Troponin, and *α*-actinin reveals the increased presence of these cardiac specific proteins after T*β*4 stimulation. (i) T*β*4 treated iPSCs show a high rate of spontaneous beating compared to untreated iPSCs in which beating was virtually absent. (*n* = 3, ^*∗*^*p* < 0.05).

To investigate the potential of Thymosin *β*4 (T*β*4) to induce cardiac differentiation in murine induced pluripotent stem cells (iPSC), we analyzed differences in the expression of a variety of transcription factors and cardiac markers at different timepoints during treatment of iPSCs with T*β*4 (Figure 1(a)). We found that during early stages of differentiation (day 4) T*β*4 significantly increased the expression of mRNA encoding the T-Bos-transcription factors Eomesodermin (Eomes) and Brachyury (T), which are indispensable for mesoderm formation in general and are known to be early mesoderm markers [17, 18] (Figures 1(b) and 1(c)). Apart from promoting the formation of early mesoderm, a treatment with T*β*4 also enhanced the expression of MesP1, the earliest marker of cardiovascular progenitors [19, 20], and NKX-2.5, which has been shown to be crucial for cardiac development [21] (Figures 1(d) and 1(e)). Similar results for the expression of MesP1 and NKX-2.5 were observed in T*β*4 treated GSES embryonic stem cells (data not shown). Finally, T*β*4 treatment after differentiation for 6 days was able to significantly increase the expression of the myocardial proteins atrial natriuretic factor (ANF) and connexin 43 (CX43) and the production of the cardiac specific proteins alpha myosin heavy chain (*α*MHC), Troponin, and *α*-actinin (Figures 1(f)–1(h)). The increased expression of contractile cardiac proteins in iPSCs via the treatment of those cells with T*β*4 resulted in a dramatic increase in contracting cardiomyocytes compared to control (Figure 1(i)).

The mode of action of T*β*4 comprises two separate physiological processes: first, T*β*4 binds monomeric G-actin, preventing it from forming or joining actin filaments via profilin, which, upon binding, favors the incorporation of actin into filaments [22]. Secondly, T*β*4 can regulate the expression of a number of serum response factor (SRF) target genes. This effect depends on the nuclear translocation of the myocardin-related transcription factor A (MRTF-A), also a G-actin binding protein [23]. An increase in actin binding to T*β*4 frees actin-bound MRTF-A leading to its nuclear translocation and SRF-binding [24, 25]. Inside the nucleus, MRTF-A acts as a transcriptional coactivator physically associating with SRF [26]. Interestingly, SRF signaling appears to be indispensable for mesoderm formation. SRF-deficient mice die during early embryonic development starting from E9.5 with impaired gastrulation and a lack of mesodermal cells [27]. Furthermore, embryonic stem cells lacking SRF are incapable of driving Brachyury (T) expression upon induction of differentiation [28]. Additionally, Nam et al. demonstrated that MRTF-A is a potent inducer of cardiac protein expression during the reprograming of human foreskin fibroblasts, increasing the amount of fibroblasts expressing cardiac troponin from 0,2% (2%) to 17% (13%) during GMT (or GHMT) induced cardiac differentiation [29]. Additionally, Thymosin *β*4 is well known to promote the differentiation of embryonic stem cells [30] and drive fibroblast to cardiomyocyte differentiation in models of myocardial ischemia [31]. Furthermore, Thymosin *β*4 has been shown to promote proliferation and reduce apoptosis under stress-conditions in mesenchymal stem cells [9], highlighting the possibility that T*β*4 might increase the pool of stem cells differentiating via proliferation and by shielding them from apoptosis. These findings point to the possibility that T*β*4 facilitates the differentiation of iPSCs to cardiomyocytes either by directly inducing differentiation, by increasing the abundance of mesodermal progenitors, which subsequently differentiate independent of T*β*4, or by protecting iPSCs during differentiation.

### 3.2. Inhibition of Thymosin *β*4 Induces Lymphatic Abnormalities in Zebrafish

**Figure 2:**
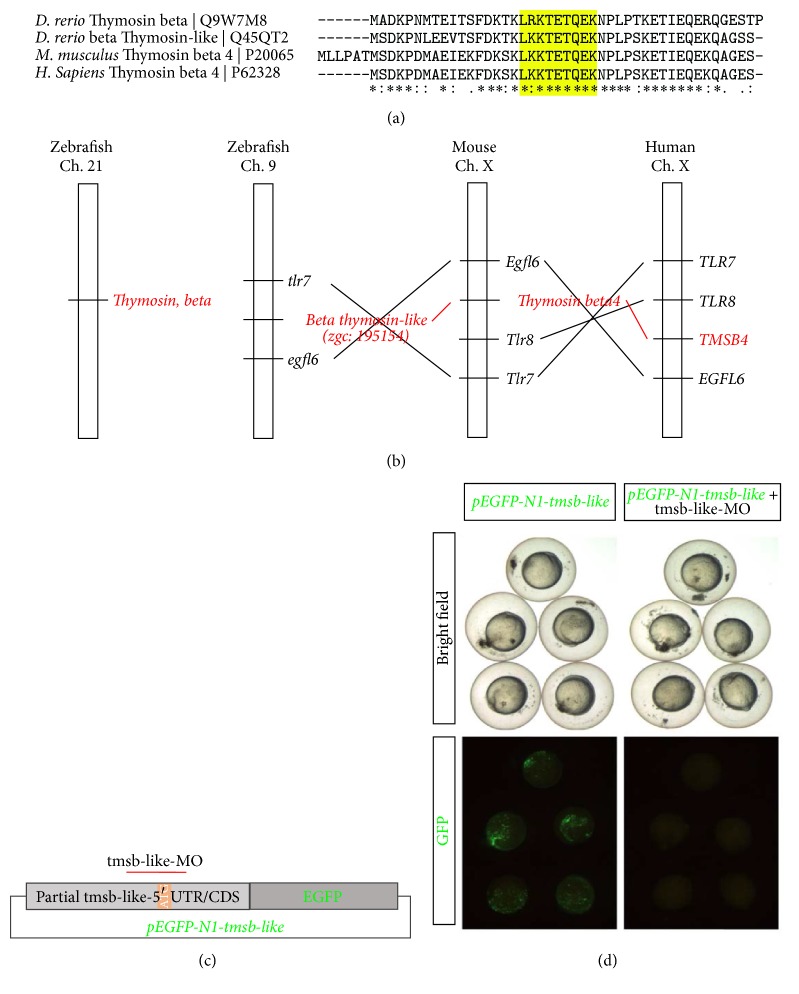
Identification of a Thymosin *β*4 ortholog in zebrafish. (a) Amino acid sequence alignment of zebrafish beta Thymosin and beta Thymosin-like and mouse and human Thymosin *β*4. The yellow box indicates the consensus actin binding motif, indicating a closer degree of homology of zebrafish beta Thymosin-like to the mammalian Thymosins than beta Thymosin. (b) The comparison of the genomic localization of beta Thymosin and beta Thymosin-like in zebrafish, in relation to the murine and human localization, shows a closer proximity of beta Thymosin-like than beta Thymosin to the mammalian Thymosin *β*4. (c) Vector used to integrate pEGFP-N1-tmsb-like in zebrafish with indicated binding site of tmsb-like morpholinos (tmsb-like-MO) used to knockdown tmsb-like in zebrafish. (d) Treatment of pEGFP-N1-tmsb-like expressing zebrafish embryos with tmsb-like-MO demonstrates high knockdown efficiency.

To investigate the role of T*β*4 during development in zebrafish, we first sought to identify which possible T*β*4 isoform in zebrafish corresponds to the human and mouse isoforms. To this end, the amino acid sequences of T*β*4 from human and mouse were aligned with the zebrafish orthologs beta Thymosin and beta Thymosin-like (Figure 2(a)). Aligning these sequences demonstrated a closer homology of the consensus actin binding sequence [32] of beta Thymosin-like to the mammalian Thymosins than that of zebrafish beta Thymosin. Additionally, the genomic localization in regard to its proximity to tlr7 and egfl6 of tmsb-like more closely resembles that of murine and human Thymosin *β*4, as demonstrated in Figure 2(b). For these reasons, we assessed the effect of a morpholino mediated knockdown of beta Thymosin-like rather than beta Thymosin. Before evaluating the effect of tmsb-like morpholino mediated knockdown on vascular development, zebrafish embryos were microinjected with GFP-tagged tmsb-like in the presence or absence of tmsb-like morpholinos (tmsb-like-MO, Figure 2(c)) to investigate knockdown efficiency. This led to a clear expression of the GFP-tagged protein, while additional injection with tmsb-like-MO blunted the expression of GFP-tmsb-like, indicating the efficiency of our morpholino mediated Thymosin beta like knockdown (Figure 2(d)). The tmsb-like morpholinos and control morpholinos were subsequently injected into Tg(fli1.egfp)^y1^ transgenic zebrafish, which express enhanced GFP under the control of the fli1 promoter in the entire vasculature to assess the embryonic development of the vasculature and lymphatic system [33].

**Figure 3:**
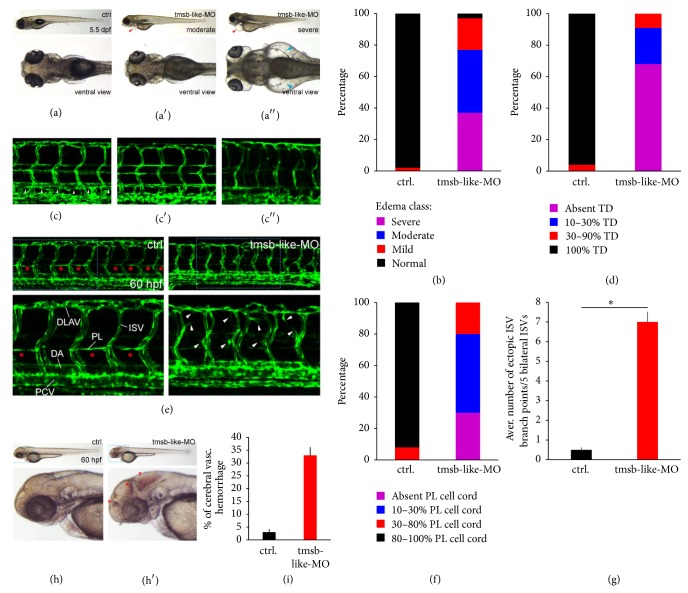
Effect of morpholino mediated tmsb-like knockdown in zebrafish. (a) Edema formation in zebrafish treated with tmsb-like-MO. Knockdown animals show moderate (a′) to severe (a′′) edema formation compared to control ((a), arrowhead), which was quantified in (b) (*n* = 123 for control-MO and 114 for tmsb-like-MO). (c) Thoracic duct (TD) formation was blunted to a moderate (c′) to sever (c′′) degree in tmsb-like-MO animals compared to proper TD formation in control animals 5.5 days postfertilization (dpf, (c), arrowhead). Results were quantified in (d) (*n* = 112 for control-MO and 103 for tmsb-like-MO). (e) Knockdown of tmsb-like leads to the aberrant formation of intersegmental vessels (ISV) displaying an ineffective hypersprouting phenotype (arrowheads) and a perturbation of parachordal lymphatic vessel generation by parachordal lymphangioblasts (PL) 60 hpf. (PCV: posterior cardinal vein, DA: dorsal aorta, and DLAV: dorsal longitudinal anastomotic vessel). (f) Presence of parachordal lymphangioblasts in 10 successive somites (*n* = 109 for control-MO and 118 for tmsb-like-MO) and (g) average number of ectopic ISV branch points in 5 bilateral ISV (*n* = 55 per group, ^*∗*^*p* < 0.05) demonstrate excessive branching and ineffective parachordal vessel formation in animals lacking tmsb-like. (h and i) tmsb-like-MO treated animals displayed a high rate of cerebral vascular hemorrhage compared to control-treated animals 60 hours postfertilization (*n* = 225 for control-MO and 204 for tmsb-like-MO, ^*∗*^*p* < 0.05).

In investigating morpholino treated zebrafish at day 5.5 postfertilization (dpf), varying degrees of edema formation were apparent in tmsb-like-MO treated animals while formation of edema was virtually absent in control-MO treated zebrafish (tmsb-like-MO with moderate edema: 40%, sever edema: 37%, Figures 3(a) and 3(b)). Since excessive edema formation is associated with malformation of the lymphatic system and defective thoracic duct formation (TD) [34–36], we investigated if a tmsb-like knockdown impairs the thoracic duct development by assessing its presence in 10 somites. These results revealed that, in tmsb-like-MO treated animals, TD formation was severely impaired at 5.5 dpf with no animals displaying TD formation in all somites and 68% showing no duct formation at all, whereas in control-MO treated animals 96% showed proper TD formation (Figures 3(c) and 3(d)). Since the thoracic duct is preceded by the emergence of parachordal lymphatic vessels (PC) that start to sprout from the embryonic posterior cardinal vein (PCV) at around 32 hours postfertilization (hpf) [37, 38], we investigated the influence of a tmsb-like KD on the pattern of PC-formation at 60 hpf by in vivo confocal imaging of Tg(fli1-egfp)^y1^ zebrafish. At this stage, the already formed main vessels (dorsal aorta (DA) and posterior cardinal vein (PCV)), which form approximately at 1 dpf [39], displayed no apparent phenotype. Intersegmental vessels (ISV) and the dorsal longitudinal anastomotic vessels (DLAV), however, seemed disorganized and displayed a hypersprouting phenotype in tmsb-like-MO treated animals compared to control (Figures 3(e) and 3(g)). Furthermore, PC-development was severely impaired as demonstrated by a reduction in parachordal lymphangioblast carrying somites (Figures 3(e) and 3(f)). The role of T*β*4 in lymphatic vessel development remains poorly understood, while the effect of T*β*4 on blood vessel maturation has been studied intensively in recent years, demonstrating the potent stabilizing effect of T*β*4 on newly formed vessels. To this end it has been shown that T*β*4, apart from inducing angiogenic sprouting via the activation of CCN1 [40], promotes the recruitment of mural cells (pericytes in particular) to the vasculature via the activation of MRTF-A and the downstream induction of the SRF target gene CCN2 [41]. Thus, the disorganization of the intersegmental vessels and the dorsal longitudinal vessels may be based on the absence of stabilizing mural cells. The hypothesis of increasing vascular destabilization in the absence of tmsb-like was further supported by the observation that tmsb-like-MO treated animals suffered from a high rate of cerebral vascular hemorrhages, a hallmark of instable vessels due to the lack of mural cells [42], while hemorrhage in control-MO treated animals was rarely seen, pointing at the presence of unstable vessels in tmsb-like-MO animals (Figures 3(h) and 3(i)). Similar phenotypic changes, an increase in vascular hemorrhage due to a reduction in mural cell coverage, have been demonstrated in mouse models of T*β*4 deficiency, indicating the importance of T*β*4 in the proper vascular development throughout vertebrate species [13]. In the case of the lymphatic system, the incompetence in lymphatic vessel sprouting seen in tmsb-like-MO zebrafish may be due to the unregulated assembly and disassembly of filamentous actin necessary for cell migration in embryonic lymphangioblasts. Another possibility is that the mechanism of sprouting and vessel maturation mediated by tmsb-like is similar in lymphatic vessels or that the deficiency in parachordal lymphangioblast sprouting stems from the disorder of ISV which are necessary guidance cues for lymphangioblast migration from the posterior cardinal vein [37].

### 3.3. Treatment of Engineered Heart Tissue with T*β*4 Improves Vascularization and Contractility

**Figure 4:**
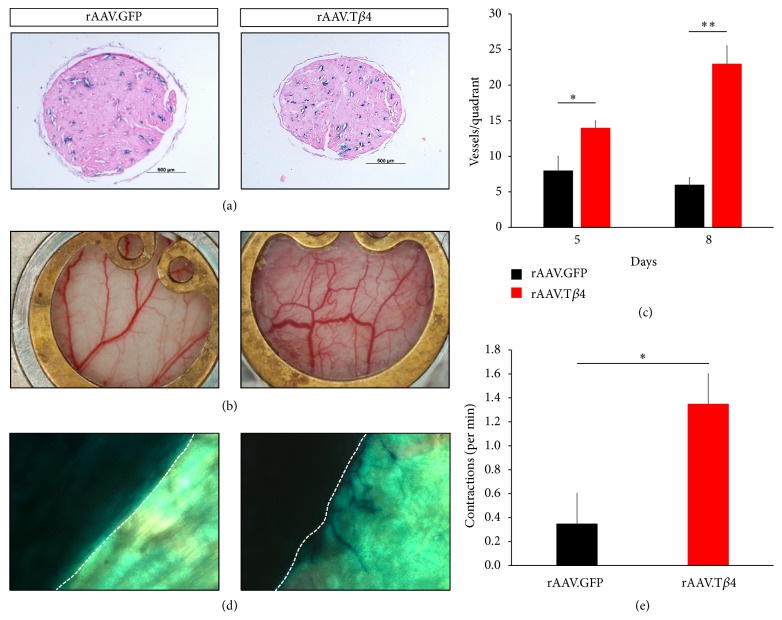
Increased vascularization and contractility in engineered heart tissue under Thymosin *β*4 treatment. (a) Cross sections of EHTs from reporter mice expressing LacZ in endothelial cells (Cdh5-CreERT2 × Rosa26-LacZ) reveal an increase in capillary density if treated with T*β*4. (b and c) AAV-mediated transfection with T*β*4 (rAAV.T*β*4, versus rAAV.GFP as control) of EHTs implanted into skinfold chambers leads to an increase of graft-vascularization, both 5 and 8 days after implantation (*n* = 4 per group, ^*∗*^*p* < 0.05; ^*∗∗*^*p* < 0.0008). (d) Images of whole skinfold chambers containing EHTs generated from LacZ reporter mice show little sprouting of graft resident endothelial cells into host tissue, while rAAV.T*β*4 treatment drastically induces angiogenic sprouting into the host tissue of skinfold chambers (dotted lines indicate the graft/host border). (e) Treatment with rAAV.T*β*4 leads to an increase of spontaneous contractions in implanted EHTs compared to rAAV.GFP control (*n* = 4 per group, ^*∗*^*p* < 0.05).

Having demonstrated that treatment of iPSCs with T*β*4 increases the abundance of functional cardiomyocytes and knowing that T*β*4 promotes vascular stability and is necessary for proper angiogenetic sprouting, we sought to investigate if a treatment of engineered heart tissue (EHT) from murine heart cells with T*β*4 promotes the vascularization of these tissue grafts. To this end, EHTs were generated from neonatal mouse heart cells of Cdh5-CreERT2 × Rosa26-LacZ mice, expressing LacZ in endothelial cells, and treated with T*β*4-peptide. Initially, cross sections of EHTs revealed a significantly higher capillary density if treated with T*β*4 (Figure 4(a)) and displayed an increase in contractile force (data not shown). Subsequently, EHTs were implanted into skinfold chambers in mice. Already macroscopically, T*β*4 treated, implanted EHTs showed a dramatic increase in vascularization (Figure 4(b)), which was confirmed by quantifying the vessels per quadrant of those EHTs 5 days after implantation (ctrl.: 8.4 ± 1.9, T*β*4 14.8 ± 1 vessels/quadrant) which became even more prominent 8 days after implantation (ctrl.: 6.4 ± 0.8, T*β*4 24.5 ± 2.4 vessels/quadrant, Figures 4(b) and 4(c)). In addition, intravenous injection of FITC-dextran demonstrated that these vessels are perfused, highlighting the functionality of these vessels (Supplementary Movie 1 in Supplementary Material available online at https://doi.org/10.1155/2017/6848271). To investigate the origin of these new vessels, we implanted EHTs from Cdh5-CreERT2 × Rosa26-LacZ mice after T*β*4 or control treatment and observed, after *β*-galactosidase staining, that the newly formed vasculature had its origin predominantly in the EHTs sprouting to the surrounding tissue of the skinfold chamber (Figure 4(d)). Lastly, to investigate the functionality of the implanted grafts, the amount of contractions was assessed and revealed a dramatic increase in the number of contractions per minute in T*β*4 treated EHTs (Figure 4(e)). Taken together, these findings implicate that the vascularization and performance of EHT can be improved by the treatment of EHTs with T*β*4. As with our results regarding iPSCs, this effect might be attributed to an increase in the fidelity of neonatal mouse cardiomyocytes during EHT production, possibly due to a decrease in apoptosis and an increase in survival, a mechanism which has been demonstrated in previous work from our group in the context of neonatal rat cardiomyocytes [43].

Combining the findings from our experiments regarding induced pluripotent stem cells and zebrafish, our results implicate that T*β*4 promotes the expansion of mesodermal progenitors, which subsequently differentiate into blood and lymphatic vessels. Those, in turn, increase the perfusion and maintenance of our EHTs. T*β*4 additionally might stabilize the cardiomyocyte phenotype of neonatal cardiomyocytes during EHT-generation by promoting the expression of cardiac proteins. Another possible mechanism, by which T*β*4 enhances EHT functionality, might be by generally promoting proliferation and inhibiting apoptosis, rendering EHTs more stable during their assembly and subsequent implantation.

## 4. Conclusion

Here we demonstrate that T*β*4 significantly increases the amount of cardiomyocyte-like cells differentiated from induced pluripotent stem cells and their respective progenitors (mesodermal cells, cardiogenic progenitors, and cardiac precursors) and that T*β*4 is required for proper embryonic development of lymphatic vessels. Furthermore, T*β*4 improves vascularization and beating capacity of EHT generated from neonatal heart cells, possibly by promoting overall proliferation, protecting from apoptosis, and inducing angiogenesis and lymphangiogenesis. These results introduce T*β*4 as a potential driver of iPSC-differentiation and EHT vascularization, improving the efficiency of generating EHTs for further preclinical and clinical use.

## Supplementary Material

In vivo fluorescence microscopy of EHT grafts implantet into skinfold chambers after injection of FITC-dextran highlights the perfusion and thus functionality of vessels within the EHT grafts.
